# Ultrasound-Guided Peripheral Nerve Blocks Performed by Orthopedic Surgeons: A Retrospective, Multicenter Study in Akita Prefecture, Japan

**DOI:** 10.1155/2021/5580591

**Published:** 2021-03-10

**Authors:** Tatsuru Tomioka, Seietsu Senma, Yuichiro Narita, Masakazu Urayama, Satoshi Yumoto, Hiroki Ito, Toshiaki Aizawa, Tsuyoshi Shirahata, Hiroshi Aonuma, Nozomi Kaga, Norimitsu Masutani, Yusuke Yuasa, Hikaru Saito, Kunio Ebata, Kentaro Ouchi, Naohisa Miyakoshi, Yoichi Shimada

**Affiliations:** ^1^Department of Orthopedic Surgery, Yokote Municipal Hospital, Akita 013-8602, Japan; ^2^Akita Hand Group, Akita 010-8543, Japan; ^3^Department of Orthopedic Surgery, Akita University Graduate School of Medicine, Akita 010-8543, Japan

## Abstract

The shortage of doctors is a societal problem, especially in rural areas such as Akita Prefecture, Japan. Therefore, it is not unusual in Akita for orthopedic surgeons to perform upper and lower limb surgeries under ultrasound-guided peripheral nerve blocks managed by the operators themselves. Multicenter studies of ultrasound-guided peripheral nerve blocks performed by orthopedic surgeons have not been reported. The purpose of this study was to clarify the safety and reliability of ultrasound-guided peripheral nerve blocks performed by orthopedic surgeons in Akita. A total of 1,674 upper extremity surgery cases operated under ultrasound-guided peripheral nerve blocks at 8 hospitals in Akita prefecture from April 2016 to April 2018 were investigated retrospectively. These blocks were performed by a total of 37 orthopedic surgeons, including senior surgeons and residents. In 321 of the 1,674 cases (19%), local anesthetics were added to the surgical field. Two cases with special factors were converted to general anesthesia. There were 2 cases of complications associated with the nerve block, but they were all transient and recovered promptly. The block site and the hospital where the block was performed showed a significant relationship with the addition of local anesthetics to the surgical site (*P* < 0.001). Surgery time, age at surgery, and surgical site showed no significant relationships with the addition of local anesthetics. The volume of the anesthetic used for the nerve block showed a significant inverse relationship with the addition of local anesthetics (*P*=0.040). Many orthopedic surgeons in Akita prefecture began to perform ultrasound-guided peripheral nerve blocks, which had a reliable anesthesia effect with no noticeable complications, whether performed by residents or senior orthopedic surgeons, and this is a useful anesthetic technique for orthopedic surgeons.

## 1. Introduction

The shortage of doctors is a societal problem, especially in rural areas such as Akita Prefecture, Japan. There are many hospitals with no full-time anesthesiologists, and the number of operations performed under anesthesiologist management is limited. Therefore, it is not unusual that orthopedic surgeons perform upper and lower limb surgeries under ultrasound-guided peripheral nerve blocks managed by the operators themselves in Akita.

The usefulness and complications of ultrasound-guided peripheral nerve blocks performed by anesthesiologists have been reported [1–4], but no multicenter studies of ultrasound-guided peripheral nerve blocks performed by orthopedic surgeons have been reported. The purpose of this study was to clarify the safety and reliability of ultrasound-guided peripheral nerve blocks performed by orthopedic surgeons in Akita.

## 2. Materials and Methods

**Figure 1:**
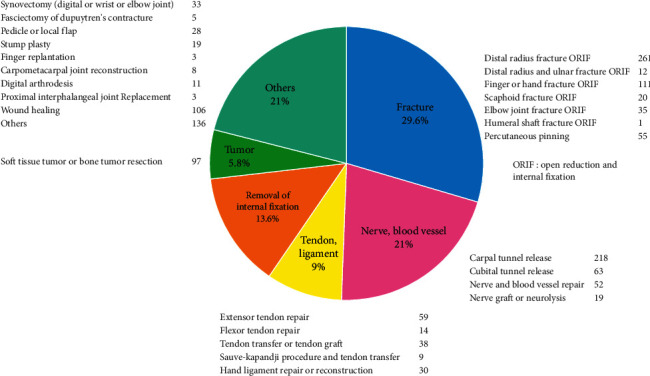
The types of 1,674 upper extremity surgery cases under ultrasound-guided peripheral nerve blocks.

A total of 1,674 upper extremity surgery cases operated under ultrasound-guided peripheral nerve blocks at 8 hospitals in Akita prefecture from April 2016 to April 2018 were investigated retrospectively. All patients were preoperatively informed by an attending doctor and agreed to undergo surgery with a peripheral nerve block. The types of operations included distal radius fracture open reduction and internal fixation (ORIF) in 261 cases, finger or hand fracture ORIF in 111 cases, elbow joint fracture ORIF in 35 cases, extensor tendon repair in 59 cases, flexor tendon repair in 14 cases, and nerve repair in 58 cases (Figure 1).

**Figure 2:**
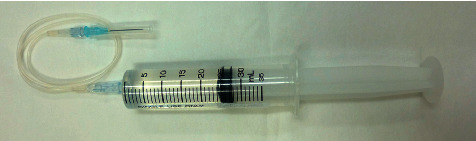
Syringe, extension tube, and needle.

The patients' average age at surgery was 58 years (range: 10–95 years). The average operation time was 67 minutes (range: 2–395 minutes). The type of block to be performed was determined by the visibility of patient's nerves, physical size, and the preference of each practitioner. Using a linear ultrasound probe, the needle was inserted across or parallel to the probe, and the needle was inserted near the nerve under ultrasound guidance. The syringe containing the anesthetics was connected to an extension tube line and 23 gauge sterile standard blunt needle (Figure 2).

**Figure 3:**
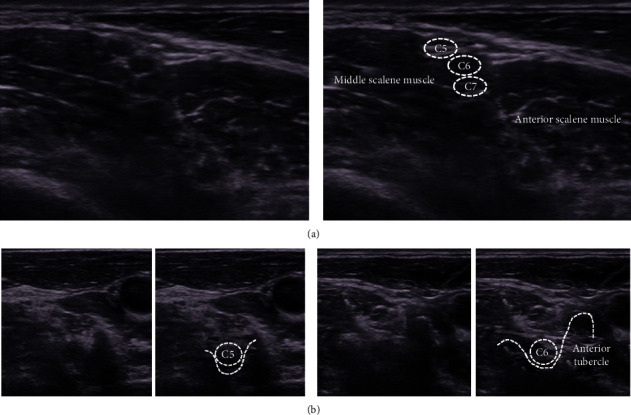
(a) In the short-axis view of the interscalene, the C5–C7 nerves depicted as straight three circular hypoechoic images between the anterior and middle scalene muscles are visualized. (b) The groove of the C5 transverse process between the anterior and posterior tubercles is U-shaped. The C6 anterior tubercle is large, so the groove of the C6 transverse process is J-shaped. The C5 and C6 nerves lead to the peripheral side through the groove of the transverse processes. Tracing the nerves depicted as circular hypoechoic images from the groove of the transverse processes to the peripheral side is useful for identifying the nerves at the interscalene.

**Figure 4:**
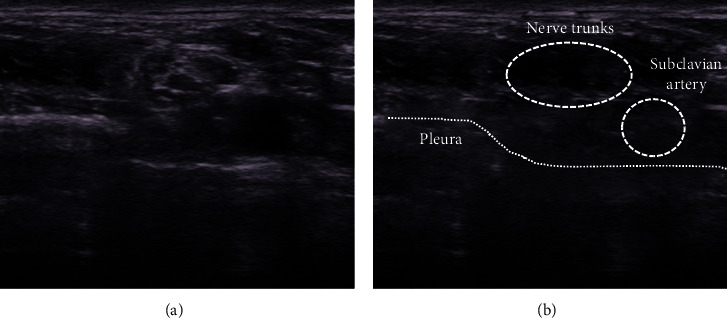
In the supraclavicular view, the linear hyperechoic pleura and hypoechoic lung are depicted, and the nerve trunks adjacent to the subclavian artery are visualized.

**Figure 5:**
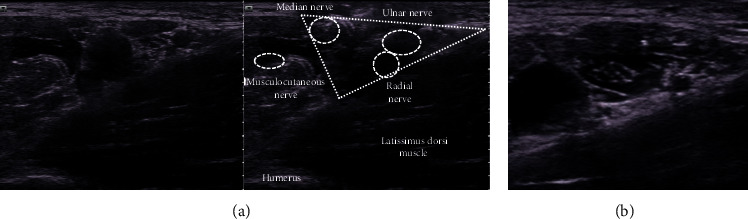
(a) In the axillary view, finding the triangle surrounded by the coracobrachialis and latissimus dorsi muscles helps identify the nerves. The median, ulnar, and radial nerves pass through inside the triangle. (b) Hypoechoic anesthetics are injected all around the nerve.

**Figure 6:**
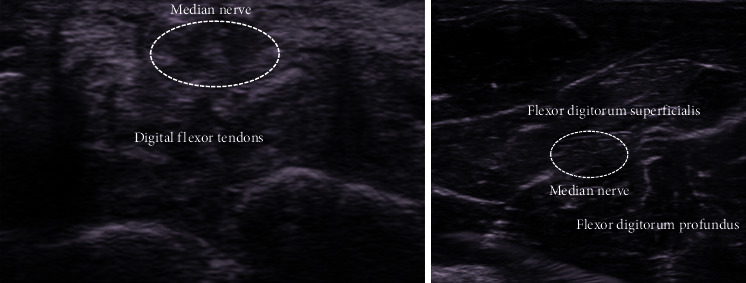
The median nerve depicted as a fascicular pattern can be identified on the superficial layer of the flexor tendons at the wrist joint level. Identifying the median nerve at the wrist level and then moving the probe to the forearm makes it easier to identify the median nerve between the flexor digitorum superficialis and flexor digitorum profundus. (a) Wrist. (b) Middle part of the forearm.

**Figure 7:**
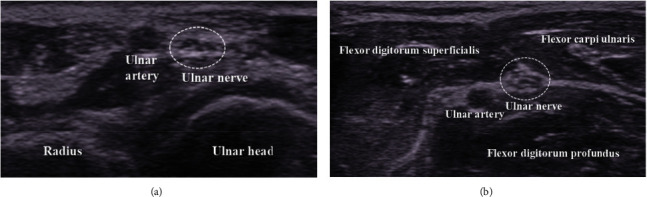
The ulnar nerve is located near the ulnar side of the ulnar artery in the range from the wrist joint to the middle part of the forearm.

**Figure 8:**
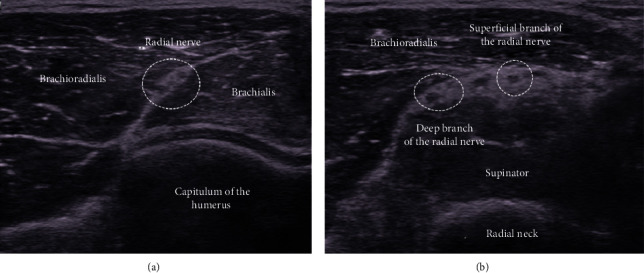
In the anterolateral short-axis view of the elbow joint level, the radial nerve is visible as a fascicular pattern between the brachioradialis and brachialis muscles. The radial nerve is divided into deep and superficial branches at the radial neck level. It is also possible to block only the superficial branch, which is the sensory branch.

The anesthetics were injected little by little about 0.5–2 ml, so as to be diffused the whole circumference of the outside epineurium and never injected intraneurally. Ultrasound-guided peripheral nerve blocks were always performed in the same image with landmark parts visualized. In the interscalene block, the C5–C7 nerves depicted as straight three circular hypoechoic images between the anterior and middle scalene muscles were visualized on the short-axis view, and each nerve was blocked (Figures 3(a) and 3(b)). In the supraclavicular block, the nerve trunks adjacent to the subclavian artery were blocked with the pleura visualized so as not to cause pneumothorax (Figure 4). In the axillary block, the triangle surrounded by the coracobrachialis and latissimus dorsi muscles was revealed. The median nerve, ulnar nerve, and radial nerve inside the triangle and the musculocutaneous nerve inside the coracobrachialis muscle were blocked (Figures 5(a) and 5(b)). In the forearm block, the median nerve, ulnar nerve, and radial nerve between the muscles were identified, and each nerve was blocked (Figures 6–8).

There were double-level blocks in some cases (17.1%). In the cases of double-level blocks, the most proximal block was considered the main block. The main blocks were the interscalene block in 86 cases, the supraclavicular block in 786 cases, the axillary block in 761 cases, and the forearm block in 41 cases. These blocks were performed by a total of 37 orthopedic surgeons, including senior surgeons and residents. In all cases, no intravenous anesthetic was used in combination. There were no cases using neurostimulators, sterile probe covers, or sterile gloves. The anesthetics used in the block were 0.25% bupivacaine, 0.75% ropivacaine, 1% mepivacaine, and 1% lidocaine, which were used alone or in combination. The volume of anesthetics in the block was 5–40 ml, with the majority of cases (87%) using less than 30 ml, depending on the weight and age of the patient. In all cases, the postoperative condition was examined by operators themselves during hospitalization and outpatient care. The operators examined for complications associated with peripheral nerve blocks.

The relationships between the addition of local anesthetics to the surgical field and the main block site, the hospital where the block was performed, and the surgical site were analyzed using the *χ*^2^ test. The relationships between the addition of local anesthetics to the surgical field and operation time, patient's age at surgery, and the amount of anesthetic fluid volume used for nerve blocks were analyzed by logistic regression analysis. All statistical tests were performed using SPSS software (SPSS 16.0J for Windows Base System, SPSS Japan Inc., Tokyo, Japan). Significance was set at *P* < 0.05.

## 3. Results

In 321 of 1,674 cases (19%), local anesthetics were added to the surgical field. Lidocaine and mepivacaine were used, but the additional anesthetic volumes were low (approximately 3–10 ml). Two cases (0.1%) were converted to general anesthesia; one case was a reduction of elbow joint dislocation with severe obesity and poor visualization of the nerves by ultrasound, and the other was a radial fracture ORIF case that was considered unsuitable for surgery under a nerve block because of psychological factors. There were two cases of complications associated with nerve blocks, namely, nausea in one case and decreased oxygen saturation in another, but both patients recovered promptly. There were no cases of persistent neuropathy or infection of the nerve block injection sites. The rate of the additional local anesthetic to the surgical field was 35% in the interscalene block, 18% in the supraclavicular block, 20% in the axillary block, and 5% in the forearm block. There was a significant relationship between the addition of the local anesthetic to the surgical site and the main block site (*P* < 0.001). The rate of addition of the local anesthetic for each hospital ranged from 6% to 34%; it was significantly different depending on the hospital in which the block was performed (*P* < 0.001).

Surgery time, age at surgery, and surgical site showed no significant relationships with the addition of the local anesthetic. On the contrary, the volume of anesthetics used for the nerve block showed a significant inverse relationship with the addition of local anesthetics (*P*=0.040).

## 4. Discussion

In a peripheral nerve block, high success rates have been reported even with conventional methods such as the transarterial technique [5] and the paresthesia technique [6], but they have the disadvantages of directly puncturing arteries and nerves. Although nerve puncture and intraneural injection were reported to, sometimes, produce temporary sensory disturbances, they did not lead to permanent neuropathy [7], whereas it was reported that neuropathy occurred in 2.8% of cases with the seeking paresthesia technique [8]. Nerve puncture and intraneural injection should be avoided as much as possible to prevent neuropathy. In a report on multiple-injection technique comparing ultrasound guidance with nerve stimulation guidance, although ultrasound guidance showed shorter time to onset of the sensory block and fewer needle insertions than nerve stimulation guidance, success rates and complication rates were similar [1]. However, there were individual variations in the locations of the nerves, and it seems easier to perform peripheral nerve blocks by identifying nerves with ultrasound [9–11]. In fact, it has been reported that the ultrasound-guided technique had less vessel puncture [12], a significantly higher success rate [13, 14], and fewer complications than the nerve stimulation-guided technique [15, 16]. However, it is necessary to be careful because there are reports that pneumothorax and nerve palsy have occurred even under ultrasound guidance [17, 18], but in the present study, despite being performed by many orthopedic surgeons, complications were only transient and mild, affecting only 0.1% of cases. Ultrasound-guided peripheral nerve blocks were performed mainly by interscalene, supraclavicular, and axillary approaches. The rate of additional local anesthetic to the surgical field was higher with the interscalene block than with other blocks, and it was thought that it was difficult to block the C8 and Th1 nerves even under ultrasound guidance. Although the number of cases of forearm blocks was small, the rate of additional local anesthetic was the lowest.

Soberón et al. reported that forearm blocks might be used as the primary anesthetic in patients undergoing hand surgery [19]. In the future, the use of forearm blocks may increase in hand and finger surgeries. The volume of the anesthetic used for the blocks in the present study was mostly 30 ml or less depending on the patient's weight and age, which was substantially less than the maximum dose. A study of the Australian and New Zealand Registry of Regional Anaesthesia reported that ultrasound guidance reduced the incidence of local anesthetic toxicity [2]. It was reported that a higher dose and concentration of the anesthetic, but not a higher volume, were associated with longer block duration [20]. On the contrary, a higher volume of the anesthetic showed a lower rate of requiring additional local anesthetic to the surgical field in the present study. It was thought that the higher volume of the anesthetic used for the nerve block could be injected more easily all around the nerve. However, it was reported that 45% of the patients who underwent an ultrasound-guided interscalene block with only 5 ml of ropivacaine developed diaphragmatic paralysis [21]; thus, it is necessary to be careful when increasing the volume of the anesthetic, especially in interscalene blocks.

The Akita Hand Surgery Group has continued to hold skill practice workshops on ultrasound-guided peripheral nerve blocks free of charge for orthopedic surgeons in cooperation with the Akita University Department of Orthopedic Surgery. As a result, ultrasound-guided peripheral nerve blocks, which were previously performed in only a few hospitals, are now performed in many hospitals throughout Akita.

However, the block site and the hospital where the block was performed showed a significant relationship with the addition of the local anesthetic to the surgical site. It may be due to the difference in the skills of the practitioners who performed the nerve block and the difference in the performance of the ultrasonic equipment used in each hospital. Therefore, in order to further improve the anesthesia success rate, it may be necessary to improve the workshop to be more accurate and comprehensible by teaching the tips and tricks for ultrasound-guided peripheral nerve blocks.

As of March 1, 2020, the total population of Akita prefecture was 960,271 and that of Akita city was 304,943; 2/3 of the population is outside of Akita city, but full-time anesthesiologists are concentrated in Akita city. Outside of Akita city, there are few emergency hospitals that have full-time anesthesiologists, and these hospitals provide surgical treatment with part-time anesthesiologists and have a manpower shortage. Under such circumstances, the number of operations that an anesthesiologist can manage for orthopedic surgery is not at all adequate. Therefore, it is not unusual that orthopedic surgeons perform upper limb and lower limb surgeries under ultrasound-guided peripheral nerve blocks performed by the operators themselves. Until now, it is unclear whether ultrasound-guided peripheral nerve blocks in Akita prefecture were performed reliably and safely.

The present study showed that cases of transition to general anesthesia were very rare, there were no noticeable complications, and the anesthesia success rate was acceptable in all hospitals.

## 5. Conclusion

As a result of continuing dissemination activities through skill practice workshops, many orthopedic surgeons in Akita prefecture began performing ultrasound-guided peripheral nerve blocks, which had a reliable anesthesia effect with no noticeable complications, whether performed by residents or senior orthopedic surgeons. This is a useful anesthetic technique for orthopedic surgeons to use.

## Data Availability

The data used to support the findings of this study are available from the corresponding author upon request.
